# Ozonation and Depolymerization of Extracellular Polymeric Substances (EPS) Extracted from a Biofilter Treating Gaseous Toluene

**DOI:** 10.3390/polym10070763

**Published:** 2018-07-12

**Authors:** Zenab Tariq Baig, Lu Meng, Prakit Saingam, Jinying Xi

**Affiliations:** Environmental Simulation and Pollution Control State Key Joint Laboratory, School of Environment, Tsinghua University, Beijing 100084, China; baigzt10@mails.tsinghua.edu.cn (Z.T.B.); menglu.555@outlook.com (L.M.); saingamp@hawaii.edu (P.S.)

**Keywords:** biofilter, biofilm EPS, ozonation, protein, polysaccharide

## Abstract

Low-concentration ozonation was developed as a novel technique to control the excess biomass in volatile organic compound (VOC) biofilters. In order to understand the reaction mechanism between ozone and biomass, the changes in properties of ozone exposed extracellular polymeric substances (EPS) were investigated in this study. EPS was sequestered from the biofilm, obtained from a biofilter treating gaseous toluene, and then it was exposed to gaseous ozone at 272 ± 22 ppm continuously for 12 h. The total organic carbon (TOC) results indicated that low concentration ozone could not mineralize the EPS to carbon dioxide (CO_2_) completely. The excitation-emission matrix fluorescence spectroscopy (EEM) results demonstrated that ozone preferred to attack the benzene ring and specific amino acid residues (such as tryptophan) on the protein chain. High performance size-exclusion chromatography (HPSEC) results confirmed that the protein molecules were depolymerized after ozone attack, while the molecular weight of polysaccharides was not much affected by ozone. During ozonation, few volatile organic compounds (VOCs), such as carboxylic acids, aldehydes, ketones, benzaldehyde and by-products of toluene, were generated, which confirms a minor change in the TOC concentration of EPS. Results revealed that low concentration ozone can reduce the molecular weight of biofilter EPS which can be a key reason for controlling biomass accumulation. Additionally, this can be used to study the composition of biofilm EPS from biofilters.

## 1. Introduction

Biofilters, either aerobic or anaerobic, are techniques designed to degrade pollutants by using diverse microorganisms growing in an aggregated form, such as biofilms or bioflocs, located or attached with filter media [1,2]. Biofilms are heterogeneous structures composed of layered microbial aggregates interlocked with extracellular polymeric substances (EPS). Biofilms have a well-defined architecture which supports an optimal environment for the exchange of genetic material between microbial cells. EPS contributes to a large part of biofilms and varies between 50% and 80% by weight. EPSs are high molecular weight polymeric organic substances, composed of mainly polysaccharides, proteins, humic acids, lipids and nucleic acid. EPS enables contrasting cell surface characteristics including morphology, strength, frictional resistance and metabolic activity [3,4,5]. These properties help to predict the behavior of biofilms in biological systems; for instance, they can lead to the abrasion of biofilms, may create frictional resistance in biofilter columns, adsorb solids from bulk liquid and may cause congestion of biofilters [6].

The composition, characteristics and applications of EPS in water, waste water and wastewater sludge, activated sludge, flocculation, dewatering and treatment is extensively researched and reported [7,8]. However, data about role of EPS in applications related to pollution control, especially in biofilters, are rather sparse and need to be explored [9]. Neilsen et al. [6] comprehensively discussed the composition of EPS extracted from biofilms in different engineered biological systems, such as in biofilters and trickling filters. Hernández et al. [10] confirmed the presence of the largest fraction of protein in EPS extracted from a methane treating biofilter and used changes in protein content as a monitoring tool to study the stress conditions. Furthermore, it was identified that protein constitutes a variety of amino acids, such as glutamic acid, aspartic acid, alanine acid and leucine acid, at a high concentration (about 40% of total proteins in EPS) [11]. Park and Novak [12] investigated the composition of amino acids in EPS and found that the structure largely depends on extraction methods. In membrane bioreactors (MBRs), EPS is reported to have complex role in fouling mechanisms [4,13]. Therefore, the performance of biofilters and the formation of biofilm is interrelated with EPS concentration and composition.

Hitherto, ozonation has been widely reported to improve the performance microbial aggregate [14,15]. The ozonation technique has been used successfully in biofilter systems as a means to control biomass inhibiting filter plugging. The effective use of ozone to reduce the biomass accumulation has been reported and validated [16,17]. The literature indicates that ozone can disrupt microbial cell membranes, oxidize lipid on cell membranes, and cause cytoplasmic efflux [18].

Studies have shown that ozone gas, not higher than 300 mg m^−3^, can effectively reduce the biomass accumulation rate in biofilters treating volatile organic compounds (VOCs) [19]. Also, low levels of ozone do not show negative/adverse effects on the metabolic activity of the microorganisms and the VOC processing performance [20]. Xi et al. [17] used 180–220 mg m^−3^ of ozone in a toluene processing biofilter and found that the biofilter clogging was effectively controlled. Additionally, a reduction in biomass accumulation and stability in biofilter performance was noticed. Thus, the relationship among ozonation, biofilm and EPS in biofilter treating waste gases is mutual and complex. However, detailed mechanism among these components is still a topic of interest. In this study, EPS was extracted from a biofilm obtained from a toluene (VOCs) treating biofilter. The main focus of the study was to investigate the effect of low concentrations of ozone on the composition and characteristics of EPS. The characteristics of EPS were analyzed in terms of their component concentration, functional groups and molecular weight (MW) distributions by using UV-Vis spectrometry, excitation emission matrix fluorescence spectroscopy (EEM), high performance size-exclusion chromatography (HPSEC) and gas chromatography-mass spectrometry (GC-MS).

## 2. Material and Methods

### 2.1. Experimental Set-Up and Operation

A lab scale biofilter was set up with a conventional layout. The biological filter tower was made up of polyvinyl chloride (PVC) with an inner diameter of 11.8 cm and bed height of 35.5 cm. The thickness of the filter bed was 20 cm, and perlite was the packing material. Activated sludge, collected from Xiaojiahe wastewater treatment plant, Beijing, China, was used as an inoculum. The concentrations of total suspended solids (TSS), volatile suspended solids (VSS) and the sludge settlement ratio were 3500–5000 mg L^−1^, 65–70% (VSS) and (solid volume per 30 min (SV30)) 25–30%, respectively, in the sampled sludge. Toluene was supplied as the processing gas to the biofilter, with the initial concentration ranging between 500 and 1300 mg m^−3^, maintaining an inflow rate of 1.5 L min^−1^. The biofilter was operated for 120 days and later analyzed for EPS properties.

### 2.2. EPS Collection and Isolation

The cation exchange resin (CER) method was used to isolate the EPS from biofilm [21]. A biofilm suspension was formed when the biofilm was separated from the filter media by gently stirring it with buffer solution. The biofilm suspension was then washed to remove impurities by using a buffer solution consisting of 2 mM Na_3_PO_4_, 4 mM NaH_2_PO_4_, 9 mM NaCl and 1 mM KCl. Maintaining the pH of buffer solution as 7, the suspension was washed by centrifuging at 2000 rpm for 15 min at 4 °C. The pretreated biofilm was transferred to an extraction flask containing CER (sodium form 732, Sinopharm chemical reagent Co., Ltd., Beijing, China) with a dosage of 60 g gVSS^−1^. The mixture of the treated biofilm and resin was stirred for 6 h at 300 rpm and 4 °C. After this, it was centrifuged twice for 10 min at 4000 rpm, and 10 min at 12,000 rpm at 4 °C to ensure the removal of CER and residual flocs. The solution containing EPS was filtered through 0.45 mm acetate cellulose membranes and used for ozonation and further analysis.

### 2.3. Ozone Experiment Setup

Ozone, generated by pumping ambient air into a UV lamp (ZW23D15W-Z436, Cnlight, Shenzhen, China), was provided to a 2.5 L ozone reactor which contained 1 L of EPS solution. The concentration of inlet ozone was controlled by a flow meter (flow rate), and both inlet and outlet ozone concentrations were measured by an ozone detector. A total reaction period of 12 h was considered in the study, where ambient air was supplied continuously to ensure sufficient reaction between EPS and ozone. A Tenax™ tube, consisting of solid sorbents (Tenax™ TA, Chrompack, Middelburg, The Netherlands, mesh 60–80), was used to collect the outlet ozone gas sample and the tube was replaced every 2 h. A stainless-steel sorbet tube (Perkin-Elmer, Waltham, MA, USA; O.D.: 6.9 mm; I.D.: 4.9 mm; length: 88.9 mm) was used for gas chromatography-mass spectrometry (GC-MS) analysis. Every one hour, a 10 mL sample was collected and analyzed for UV-Vis spectrometry, EEM and HPSEC analysis as per standard procedures. All the experiments were done in triplicate to ensure accuracy.

### 2.4. Analytical Methods

#### 2.4.1. Analysis of Polysaccharides, Protein and TOC

Polysaccharides and protein are major components of EPS. The anthrone method, with glucose as a standard, was used to analyze the polysaccharides content of biofilm EPS [22], while the protein content was measured by using the Lowry method with bovine serum albumin (BSA) as a standard [23]. The total organic carbon (TOC) concentration of EPS was measured by a TOC analyzer (TOC-VCPH, Shimadzu, Kyoto, Japan), while a UV spectrophotometer (UV-2401, Shimadzu, Kyoto, Japan) was used to measure the UV-Vis absorption spectra.

#### 2.4.2. Analysis of the EEM Spectra of EPS

In this study, the EEM spectra of EPS were measured by using a luminescence spectrometer (F-7000, Hitachi, Chiyoda-ku, Japan) with subsequent scanning emission spectra ranging from 240 to 600 nm at 1 nm increments and excitation wavelengths from 220 to 450 nm at 5 nm increments. The scanning speed was 30,000 nm min^−1^, and both excitation and emission slits were 5 nm. In order to eradicate second order Raleigh light scattering, a 290 nm emission cutoff filter was used. EEM data were handled by software Origin 7.5 (Origin Lab Corporation, Northampton, MA, USA). The spectra of Milli-Q water were used as blanks. EEM spectra were demonstrated as having the elliptical shape of contours.

#### 2.4.3. Analysis of Molecular Weight Distribution of EPS

HPSEC (LC-20 AD, Shimadzu, Kyoto, Japan) equipped with a PDA detector (SPD-M20A, Shimadzu, Kyoto, Japan) and RID-10 detector (Shimadzu, Kyoto, Japan) with a TSK column (G3000PWXL, TOSOH, Tokyo, Japan) was used to measure the molecular weight distribution in EPS. Column temperature was maintained at 40 °C and it was calibrated using polyethylene glycol (PEG) as a standard. Milli-Q water was used as the mobile phase at a flow rate of 0.5 mL min^−1^ and injection volume was 100 mL. In HPSEC chromatographs, UV254 signals intensity aims to detect protein content shown in red color line, while black line shows RID signals intensity, representing the polysaccharide components in EPS. The principle of HPSEC based on difference in retention times/intensity of the molecules which is characterized by size and concentration. Small molecules can penetrate the column of HPSEC chromatography quickly, while very large molecules penetrate the column slowly [24].

#### 2.4.4. Analysis of VOCs from the Ozone Reactor

Volatile organic compounds (VOCs) produced during the ozonation process were identified by thermal desorption unit ATD 650 (Perkine Elmer Corp., Shelton, CT, USA) combined with a GC-MS-QP2010 Plus gas chromatograph (Shimadzu, Kyoto, Japan) equipped with a DB-5MS 60 m × 0.32 mm × 0.5 µm column (Agilent Technologies, Santa Clara, CA, USA). ATD 650 was connected to GC-MS through a valve and a transfer line maintained at 210 and 215 °C, respectively. The conditions employed for the thermal desorption system were as follows: the desorption temperature and time were 250 °C and 10 min, respectively, the trap heat and cool temperature were 280 and −25 °C, respectively. Argon gas was used as the carrier gas with a flow rate of 1.5 mL min^−1^. The sequential programmed linear temperature was used in ATD 650 system as; (1) 5 °C min^−1^ to 120 °C (hold for 2 min); and (2) 10 °C min^−1^ to 260 °C (hold for 5 min). The ion source temperature was maintained at 200 °C and GC oven temperature was 40 °C (hold for 2 min).

## 3. Results and Discussions

### 3.1. EPS Components Profile

The EPS solution extracted from the biofilm was a pale yellow clarifying liquid with neutral pH (7.4). The contents of polysaccharides and protein in EPS were 2.0 and 18.9 mg gVSS^−1^ in VSS and are similar to content values found in the existing literature, indicating that same extraction method was used and it was found effective [25]. In addition, the value of TOC (27.2 mg gVSS^−1^) in EPS was significantly higher compared to other components due to the presence of high carbon content in the form of toluene metabolites. Additionally, protein and polysaccharides may have contributed to the TOC because of their organic nature.

### 3.2. Utilization of Ozone

**Figure 1:**
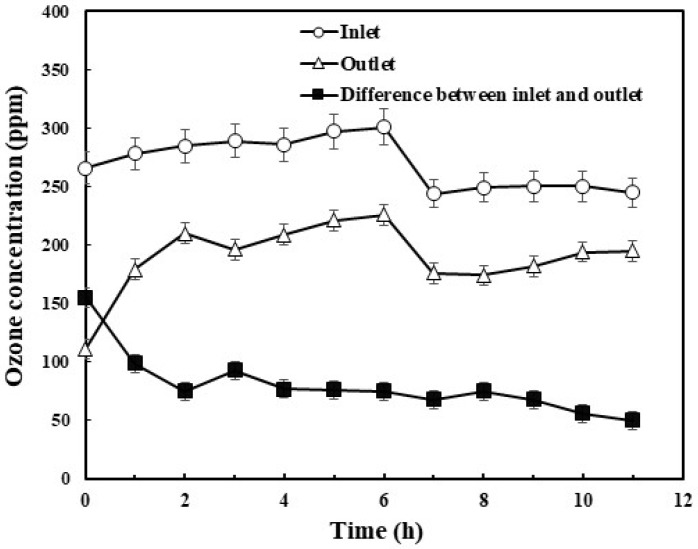
Inlet and outlet ozone concentrations at different intervals during extracellular polymeric substances (EPS) ozonation.

Figure 1 represents the inlet and outlet concentration of ozone consumed during the ozonation process. The initial inlet and outlet concentrations of ozone were 272 ± 22 ppm and 155 ppm, respectively. During the first 4 h, the ozone consumption rate was significantly higher, indicating a potential reaction between EPS and ozone. After 4 h, the difference in ozone inlet and outlet concentration was noticed to be 50 ppm, indicating that EPS is reacting slowly with ozone and attaining equilibrium.

### 3.3. Effect of Ozone on Components of EPS

**Figure 2:**
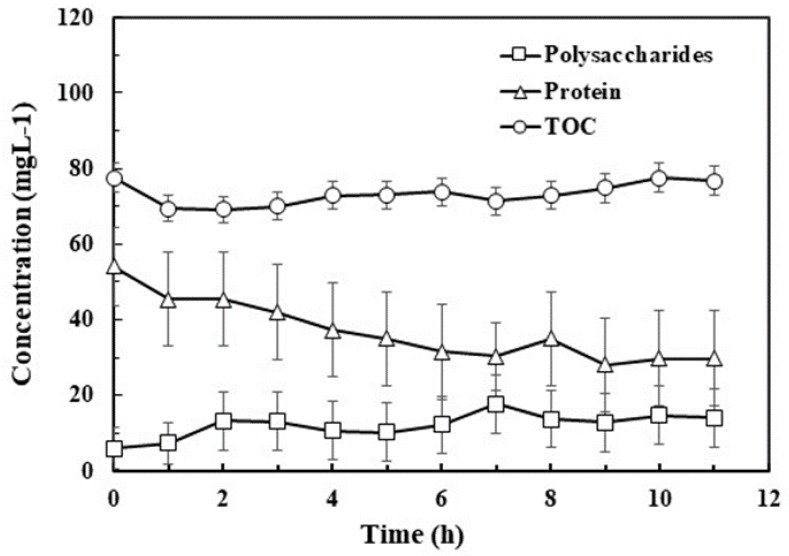
Protein, polysaccharides, and total organic carbon (TOC) concentrations at different intervals in EPS solution during the ozonation process.

A variation in the polysaccharide, protein and TOC content was observed in EPS once low concentrations of ozone were injected (Figure 2). Polysaccharide concentrations slightly changed from 6 to 14 mg L^−1^ over the 12 h reaction period. Meanwhile, protein experienced a two-stage ozonation process; in the first stage, from 0–8 h, protein content decreased by 35% reflecting a sharp reduction in the protein part. In the second stage, 8–12 h, the reaction of protein with ozone reached equilibrium at 30 ± 3 mg L^−1^ with no further substantial change. The shift in the TOC content of EPS with reaction time was ambiguous and remained at 73 ± 3 mg L^−1^. Briefly, polysaccharide and protein concentration corresponded to ozone consumption pattern, except TOC that remained unaffected because of incomplete organic mineralization, demonstrating the high utilization rate of ozone in first 4 h deflating the polysaccharides and protein.

Beside other polymeric clusters, EPS contain sites that are reactive to ozone. Ozone reacts faster with several functional groups because of its selective and strong oxidant nature [26]. Therefore, ozone can easily oxidize the EPS into different component units. Pan et al. [27] reported that polysaccharide chains consisting of glycosidic bonds can be easily cleaved into anomeric C–H bonds after ozone attack. The breakdown of hydrogen trioxide results in aldonic acid lactone fragments, which are responsible for smaller molecular chains of polysaccharides or oligosaccharides [28]. Small molecules (by-products of polysaccharides) after ozonation were detected as polysaccharides by the anthrone colorimetric method, and this was due to the presence of hexose and hexuronic acid. The main change brought by ozonation appeared to be decrease of molecular weight of EPS. In addition to EPS break-up, ozonation at a low concentrations also modified polymer’s chemical properties such as cracking, fragmentation and changes in surface properties. [29].

Protein content was determined by the folin phenol method, and the folin reagent reduced tyrosine and tryptophan residues in the protein. It has been validated in a study that ozone oxidation mainly occurs in tyrosine, tryptophan, histidine, cysteine and methionine residues for polypeptides and proteins [30]. Another study showed that aromatic amino acids such as phenylalanine and tryptophan phenylalanine react with ozone and have high reactivity [31]. Thus, one can conclude that tyrosine and tryptophan in protein in EPS are greatly reduced due to the significant consumption of ozone in the early period (first 4 h).

### 3.4. Influence of the Ozonation Process on Molecular Weight (MW) Distributions and Functional Groups of EPS

#### 3.4.1. Molecular Weight (MW) Distribution

**Figure 3:**
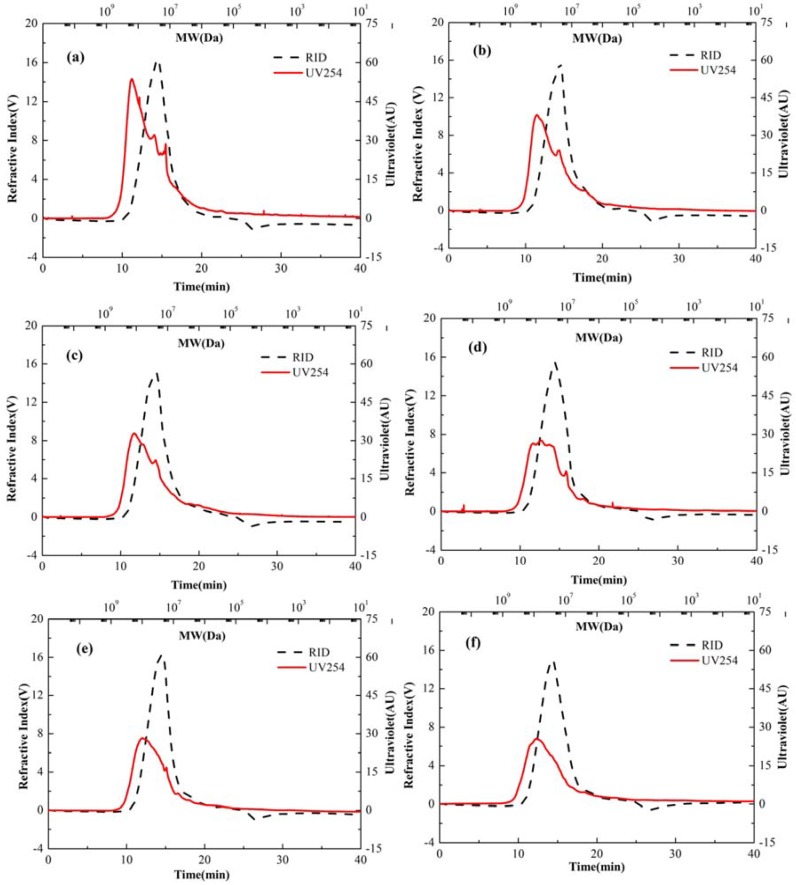
Molecular weight distribution of EPS at ozonation times of (**a**) 0 h; (**b**) 1 h; (**c**) 2 h; (**d**) 4 h; (**e**) 6 h; (**f**) 12 h.

HPSEC chromatographic analysis was carried out in order to analyze the molecular weight distribution of EPS before and after ozonation (Figure 3). The shift in the molecular weight distribution of EPS components can be analyzed by the intensity and retention time of peaks in HPSEC chromatographs during ozonation process. Figure 3a shows that the UV254 (protein) peak and RID (polysaccharides) peak appeared at around 11 and 15 min of retention time at the start of the ozonation process (0 h). The retention time of protein peak increased to 12.5 min but polysaccharide peak did not show any remarkable change at the end of ozonation process (12 h). The intensity of the protein peaks decreased as ozonation process progressed as shown in Figure 3b–e, while the intensity of the polysaccharide peaks remained constant. All the results above indicate that protein could be somewhat disintegrated into small molecules after ozone attack, while the molecular weight of polysaccharides was not affected by ozone.

There could be two reasons that EPS molecular weight reduction was not significant. Firstly, the exposure time of ozone was not enough (12 h) for EPS collected from biofilter biofilm that could lead to the complete destruction of polymers as they have a high molecular weight and are long chain macromolecules. Secondly, EPS was extracted from the toluene treating biofilter and benzaldehyde (by-product of toluene) can act as an interfering compound which can further inhibit or affect the biofilm EPS depolymerization.

#### 3.4.2. UV-Vis Absorption Spectra of EPS

**Figure 4:**
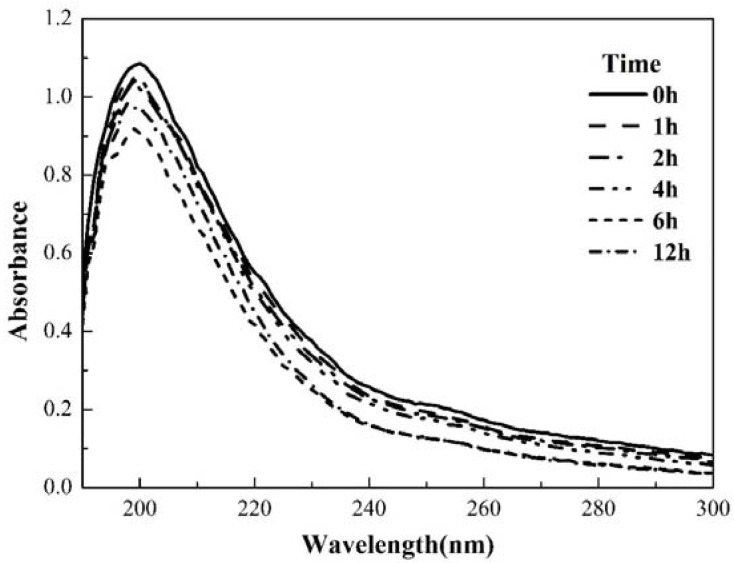
Biofilm EPS UV-Vis absorption spectra during the ozonation process.

Figure 4 represents the UV-Vis spectra of EPS with different time intervals. Results from EPS showed one absorption peak at 220 nm before reaction with ozone (0 h). The maximum absorption wavelength shift specifies the presence of a benzene ring with a conjugated double bond in EPS. During the ozonation process, the wavelength and intensity of peaks in UV-Vis spectra changed significantly with time. The absorption peak at 220 nm remained uniform with declined intensity illustrating the attack of ozone on benzene ring structure. Conclusively, the degradation of aromatic compounds such as benzene in protein has been observed.

#### 3.4.3. EEM Spectra of EPS

**Figure 5:**
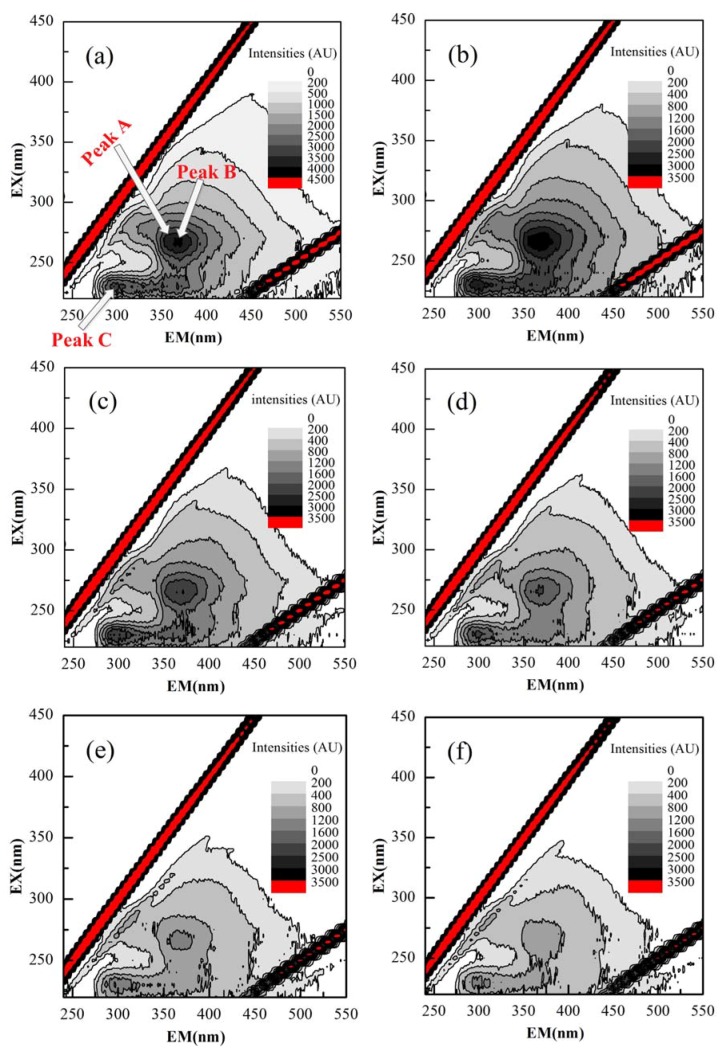
Excitation-emission matrix (EEM) fluorescence spectra of EPS at ozonation time of: (**a**) 0 h; (**b**) 1 h; (**c**) 2 h; (**d**) 4 h; (**e**) 6 h; (**f**) 12 h (Peaks A & B: Soluble microbial by-products, Peak C: Aromatic protein (tryptophan)).

The position and intensity of peaks in three-dimensional EEM fluorescence spectra revealed the information about functional groups present in EPS. A total of three peaks from the EEM fluorescence spectra of EPS have been identified at 0 h (before reaction), shown in Figure 5. Peak A was attributed to the soluble microbial by-products (SMP) with an excitation/emission wavelength (Ex/Em) of 280/310 nm. Peak B at an Ex/Em of 270/370 nm demonstrated soluble microbial by-products (SMP), while the position of peak C elucidated the occurrence of tryptophan-like aromatic protein with an Ex/Em of 225/300 nm [32]. After some interval, the intensity of peaks B and C reduced sharply and almost disappeared, highlighting that ozone was consumed to eliminate a large amount of fluorescent substances in EPS, mainly SMP and tryptophan-based aromatic proteins.

#### 3.4.4. VOC Production under Ozone Exposure

VOCs produced during the ozonation process were detected by gas chromatography (GC-MS). The oxidative products of the export gas (VOCs) were mainly carboxylic acids, aldehydes, ketones and toluene as reported in other studies. These studies revealed the reaction mechanism between ozone and natural organic matters (NOM), humic acid and protein [33,34,35]. It has been reported that ozone attacks hydroxyl groups at C2, C3, and C6 positions in polysaccharides to produce carbonyls, while carbonyls are easily degraded to carboxyl groups under ozone exposure [36].

In this study, toluene was a primary carbon source for the biofilm and it was oxidized into benzaldehyde and other metabolites before and during the ozonation process. It was difficult to quantify the VOCs produced, but the transformation of organic carbon into carboxylic acid, ketones, aldehydes and toluene by-products could be a possible reason for the reduction of TOC in EPS. Thus, conclusions can be made that ozone reacts with EPS to form carboxylic acid, ketones, aldehydes, and by-products of toluene and other small molecules, justifying the idea that most of these compounds are volatile and readily biodegradable.

## 4. Conclusions

This study verified that the application of a low concentration of ozone injection is a promising method to control the biomass accumulation in terms of molecular weight reduction in toluene-treating biofilters and can enhance their performance in long-term operation. Results indicated the considerable variation in the protein and polysaccharide content of EPS, at around 35% and 137%, respectively, after continuous injection of gaseous ozone. EPS was not completely mineralized to CO_2_ after ozonation, as indicated by total organic carbon (TOC) analysis. At the end of the ozonation process, the molecular weight of EPS was reduced, but the difference was minor compared to previous studies. The reduction in molecular weight can be deduced from the fact that the ozone exposure time was not enough to effectively lower the biomass accumulation. It was also inferred that toluene produced some metabolites/by-products such as benzaldehyde that can interfere with the ozonation process. To conclude, these results can provide details about the effect of low concentrations of ozone on characteristics of EPS and represents a baseline study to further understand the reaction mechanism between ozone and EPS.
